# Reply: Occlusion Myocardial Infarction: From Theoretical Concept to Practical Therapy

**DOI:** 10.1016/j.jacadv.2024.101550

**Published:** 2025-01-08

**Authors:** Jesse T.T. McLaren, José Nunes de Alencar, Emre K. Aslanger, H. Pendell Meyers, Stephen W. Smith

**Affiliations:** a University Health Network, Toronto, Ontario, Canada; b Instituto Dante Pazzanese de Cardiologia, São Paulo, Brazil; c Marmara University Pendik Training and Research Hospital, İstanbul, Turkey; d Carolinas Medical Center, Charlotte, North Carolina, USA; e Department of Emergency Medicine, Hennepin County Medical Centre, and University of Minnesota School of Medicine, Minneapolis, Minnesota, USA

We thank Dr Thomas and Dr Zoni and colleagues for their replies to our proposed paradigm shift from ST Elevation Myocardial Infarction (STEMI) to occlusion MI (OMI).^1^

We are all in agreement that the STEMI/non-STEMI dichotomy is an outdated paradigm that does not capture the complexities of acute coronary occlusion and leads to preventable delays to reperfusion and higher mortality. Such agreement on the foundations of the current paradigm reflects its current crisis, and debating the new paradigm shift is a welcome development in emergency cardiology. How, then, should acute MI be better classified both conceptually and practically?

Dr Thomas calls for a conceptual reclassification of coronary artery disease to reflect acuity (acute vs chronic) and severity (total, partial, and mixed).^1^ We agree with Dr Thomas’s perspective that this disease is not a dichotomous paradigm but a spectrum with multiple shades of gray. It is important to discuss and integrate a terminology that encompasses partial occlusions, spontaneously reperfused occlusions, vasospasm-induced occlusions, and other such conditions into the near future of this new paradigm. Our goal is to promote a paradigm shift that affects patient management at the point of initial contact, enabling timely interventions to minimize ongoing infarction.

However, we do agree on the need for a conceptual framework that reflects the underlying dynamic pathology of acute coronary occlusion. Dr Thomas prefers the term “obstruction” (an “abstract clinic-pathologic concept”) rather than “occlusion” (which represents “angiographic findings”). OMI is neither an abstract concept nor an angiographic finding: “OMI is defined conceptually as acute coronary occlusion or near occlusion with insufficient collateral circulation, such that downstream myocardium will undergo imminent necrosis without reperfusion. OMI has been used as the outcome definition for many studies of electrocardiogram (ECG) interpretation over the past 10 to 15 years.”^2^ This definition includes angiographic culprit, TIMI flow, peak troponin level, and echocardiography when angiography is not performed. Future studies of OMI diagnosis will include computed tomography coronary angiogram, intravascular ultrasound, and optical coherence tomography, as well as cardiac magnetic resonance imaging.

Dr Zoni and colleagues call for a therapeutic reclassification of acute coronary syndromes based on whether or not immediate reperfusion is required. We are aware and highly enthusiastic to see that the European Society of Cardiology’s acute coronary syndrome guidelines unify STEMI and non-STEMI, reflecting precisely this spectrum view of the syndrome. When Dr Zoni and colleagues propose that the syndrome should be classified into cases requiring immediate reperfusion and those that do not,^3^ we fully agree. In fact, our proposed classification defines OMI as described above, thereby identifying lesions amenable to reperfusion. We agree that a time-sensitive, clinically emphasized classification should be the future of this paradigm.

To emphasize, the OMI paradigm is neither defined by the ECG nor is it simply a reflection of angiographic findings. In our classification, OMI represents the underlying process of coronary artery thrombosis, regardless of the diagnostic modality used to identify it. By decoupling the disease name and classification from a single ECG feature (ST-segment elevation), the OMI paradigm focuses on the actual pathophysiological event, assessed clinically with all available information, not solely on angiographic findings.

A new understanding of these pathologies will bring advances in clinical, ECG, and echocardiographic findings to guide emergent reperfusion, followed by studies of outcomes to promote new research and quality improvement. Leveraging these advances, artificial intelligence is now being trained to identify OMI on ECG with high accuracy,^4^ which will include the clinically important differentiation between occlusion, reperfusion, nonspecific subendocardial ischemia, and none of the above.

In conclusion, we believe we are all discussing the same objective and striving for better patient outcomes through a paradigm that is not dichotomous, is clinically focused, does not rely on a single test, and can bring renewed diagnostic and therapeutic advancements for this disease. We are ready to join forces to change this paradigm.
